# The Impact of Audio Book on the Elderly Mental Health

**DOI:** 10.18869/nirp.bcn.8.5.361

**Published:** 2017

**Authors:** Fereshteh Ameri, Naser Vazifeshenas, Abbas Haghparast

**Affiliations:** 1. Scientific Resources Management, Central Library and Archives, Shahid Beheshti University of Medical Sciences, Tehran, Iran.; 2. Neuroscience Research Center, School of Medicine, Shahid Beheshti University of Medical Sciences, Tehran, Iran.

**Keywords:** Mental disorders, Mental health, Bibliotherapy, Audio book

## Abstract

**Introduction:**

The growing elderly population calls mental health professionals to take measures concerning the treatment of the elderly mental disorders. Today in developed countries, bibliotherapy is used for the treatment of the most prevalent psychiatric disorders. Therefore, this study aimed to investigate the effects of audio book on the elderly mental health of Retirement Center of Shahid Beheshti University of Medical Sciences.

**Methods:**

This experimental study was conducted on 60 elderly people participated in 8 audio book presentation sessions, and their mental health aspects were evaluated through mental health questionnaire (SCL-90-R). Data were analyzed using SPSS 24.

**Results:**

Data analysis revealed that the mean difference of pretest and posttest of control group is less than 5.0, so no significant difference was observed in their mental health, but this difference was significant in the experimental group (more than 5.0). Therefore, a significant improvement in mental health and its dimensions have observed in elderly people participated in audio book sessions. This therapeutic intervention was effective on mental health dimensions of paranoid ideation, psychosis, phobia, aggression, depression, interpersonal sensitivity, anxiety, obsessive-compulsive and somatic complaints.

**Conclusion:**

Considering the fact that our population is moving toward aging, the obtained results could be useful for policy makers and health and social planners to improve the health status of the elderly.

## 1. Introduction

The aging population process has already begun in our country. It is predicted that in 20 years (from 2006 to 2026), the mean age of the population will increase by 10 years, and around 21%–25% of the population will be old by 2051 (Mirzaei & Shams Ghahfarokhi, 2007). Thus, the old people’s health care should be considered more and more in Iran. Along with rising the elderly population, their health problems, especially mental health problems becomes more important (Bowling & Windsor, 1997). After retirement, social relations of the elderly people decrease and their emotional complications increase. In this regard, the studies on the factors affecting the promotion of qualitative and quantitative of elderly life are essential (Mirzaei & Shams Ghahfarokhi, 2007). Mental health professionals should investigate the treatment of mental disorders, especially depression (Watt & Cappeliez, 2000).

In psychiatry and clinical psychology, bibliotherapy is known as an adjunct to individual treatment and guidance to resolve the personal problems. It is one of the techniques of psychotherapy and must be used according to the therapist’s advice purposefully and in an organized way. Reading books is one of the tools that improve mental disorders. Some elderly are unable to read alone due to various reasons like aging, poor eyesight, or illiteracy. Audio books are useful for people who lacks the ability to read or enough time to study. It is the best choice to improve the quality of lives and protect the social health (Henasabzadeh Esfahani, 2011). Father of American Psychiatry in the 19th century, Benjamin Rush, was the first psychiatrist who used reading as a part of the treatment process. Also, the American Library Association was the first professional organization that tried to promote bibliotherapy by expanding hospital and prison libraries (Al Yasin, 2000).

Today, in developed countries, bibliotherapy is used frequently and effectively in social environments such as hospitals, schools, prisons, and rehabilitation and counseling centers to treat some common psychiatric disorders such as depression (Baloochzeraatkar, 2004). The purpose of the bibliotherapy includes giving individual tips in three areas: 1. Stay safe from disease (prevention); 2. Problem solving (treatment); and 3. Personality development and emotional maturity (concept of improvement) (Henasabzadeh Esfahani, 2011).

Currently some people, who need psychiatric services and counseling, have little desire to visit mental health specialists and even in some cases, despite their serious or chronic condition, refuse to visit a doctor. The use of psychotherapy techniques, particularly bibliotherapy can be the perfect solution to improve their mental problems such as depression and anxiety (Henasabzadeh Esfahani, 2011). Since the problem of aging can be studied from various aspects, regarding the importance of mental health, especially in the elderly, this study examined whether audio books affect various aspects of mental health such as interpersonal sensitivity, somatization, obsessive-compulsive, depression, anxiety, hostility, phobic anxiety, paranoid ideation, psychosis and consequently improve mental health of the retired elderly.

## 2. Methods

The study population comprised all retired people in the center of Shahid Beheshti University of Medical Sciences in 2016. The sample consisted of 30 elderly volunteers in the experimental group and 30 elderly volunteers in the control group. The elderly were selected using simple random sampling method. The criteria for participating of the elderly people were as follows: Age range: 65 to 80 years; Sufficient interest in participating in the study; Having adequate literacy; Having ability and patience to sit in audio book courses; and Participating at least in 5 sessions of audio book courses.

The experimental and control groups are organized based on individual matching. Membership in groups was random without the interference of the researchers. We tried to match all cases in these groups except for participating in audio book workshops (independent variable) to be sure that the probable difference in mental health of the elderly was due to the effect of attending the audio book workshops. The audio book course was conducted for 1 month, 2 days per week, in 8 one-hour sessions. After selecting samples, mental health standard questionnaires (SCL-90-R) were distributed among both control and experimental groups and then courses of audio book were held for the experimental group. For selecting books, researchers tried to choose the optimal and correct choice based on the interest of the elderly. These books were provided from production and distribution centers of audio books such as Rasa Book, Avanameh, and Audiolib.

The most important books were in the followings categories: Poetry and literature: *Shahnameh, Masnavi Manavi, Divan-e-Hafez* and *Divan-e-Sa'di*; Psychology: C*onquer the summit of success without limbs by Nick Vujicic, The power of expression (Talk to win) by Brian Tracy*; Religion: *The Holy Quran*; Fiction: *Iran’s constitutional history.*

Audio book workshops were held for the elderly as a group in 8 one-hour sessions. The elderly in the first 20 minutes of session listened to the psychology audio book and after 5 minutes of rest, they listened to the audio book in poetry for 10 to 15 minutes. Poetry books with poems of great poets such as Hafez, Ferdowsi and Sadi were selected and tried to select lyrics with different contents. The last 20 minutes of audio book session was held with different contents, for example, in religion or fiction. We tried to choose the content of the first 20 minutes of session on a specific topic in all 8 sessions, so that in addition to being informative, arouse curiosity and interest of the elderly to participate in the next sessions. Unfortunately, in some sessions, some elderly people for reasons such as personal problems or illness did not attend, so in order to compensate their absence, they were asked to listen to audio book that was given to them individually. After completing the sessions, the SCL-90-R questionnaires were distributed among them to determine the effects of audio books.

Finally, the gathered data were analyzed and compared with pretest results. SCL-90-R has been designed to measure the psychological aspects of physical and mental illnesses and has both validity and reliability as a standard questionnaire. Subjects’ responses to each of its 90 questions is rated on a discomfort scale of 5 from “never” to “strongly.” This test covers 9 symptoms of mental illnesses: somatic complaints, obsession and compulsion, interpersonal sensitivity, depression, anxiety, aggression, phobic anxiety, paranoid ideation, and psychosis. Various studies indicated the high reliability of this questionnaire (Yaghoobi, 2008).

To measure the reliability of the research data collection tool, the Cronbach α coefficient was used which the results are presented in Table 1. This reliability coefficient refers to internal consistency of the questions; i.e., the test questions are, to what extent, have cross correlation with each other. The Cronbach α coefficient was calculated more than 7.0 for all parts of the questionnaire. However, the reliability has been verified and does not take corrective action in this regard. The Cronbach α was calculated using the following equation:

$$a=\frac{k}{k-1}(1-\frac{\sum_{i=1}^{k}S_{i}^{2}}{S_{t}^{2}})$$

**Table 1.** The reliability of the research questionnaire.

**Variables**	**Observations Number**	**Questions Number**	**Cronbach α Coefficient**
Interpersonal sensitivity	30	9	0.858
Somatic complaints	30	12	0.743
Obsession-compulsion	30	10	0.798
Depression	30	13	0.727
Anxiety	30	10	0.878
Aggression	30	6	0.926
Phobic anxiety	30	7	0.891
Paranoid ideation	30	6	0.885
Psychosis	30	9	0.906
Mental health	30	90	0.955

The obtained data were analyzed using SPSS 24. The statistical tests comprised descriptive statistical methods, values of central tendency (mean, median, and mode), dispersion (standard deviation, range, minimum and maximum observation) and the distribution (skewness and kurtosis), pie chart, bar chart, line chart and rank histogram of frequency and inferential statistics methods, 1-sample Kolmogorov-Smirnov test, Independent samples t test, paired samples t test, analysis of covariance (ANCOVA) and Friedman test. It is worth noting that the significance level for all tests was considered as 0.05.

## 3. Results

The results of this study are presented in two parts; the first part is descriptive statistics and the second part is inferential statistics where the study questions and the proposed hypotheses were analyzed by 1-sample Kolmogorov-Smirnov, Independent samples t test and paired samples t test, so that the results of this research help researchers in achieving the study objectives.

Characteristics of study subjects including gender, marital status, age, education level, are presented in Table 2. According to Table 2, among 60 samples, female respondents (61.7%) are more than male ones. Study participants without spouse (53.3%, due to death or divorce) are more those who had spouse. The number of respondents between 60 and 70 years (65%) are more than respondents in other age groups and the number of 60 years respondents (13.3%) is less than other respondents. The participants with MS degree (48%) are more than respondents with the other degrees of education and the respondents with diploma (1.6%) had the lowest number.

**Table 2.** Frequency and percentage of samples' characteristics.

**Samples’ Characteristics**		**No.**	**%**
Gender	Male	23	38.3
	Female	37	61.7
Marital status	Without spouse	32	53.3
	With spouse	28	46.7
Age	60 years	8	13.3
	Between 60 and 70 years	39	65.0
	More than 70 years	13	21.7
Education level	Diploma	1	1.6
	Associate degree	4	6.6
	Bachelor degree	26	43.3
	Master degree	29	48.5

### 3.1. Samples’ favorite types of books

**Figure 1.**
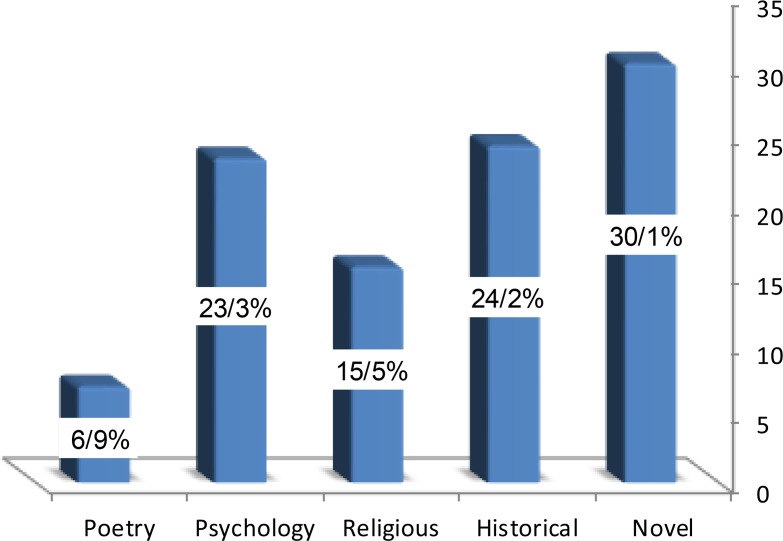
Frequency of the studied samples based on the respondents’ favorite book type.

According to Figure 1, of 103 favorites registered, 30.1% of the participants were interested in novels, 24.2 % in history books, 15.5% in religious books, 23.3% in psychology books, and 6.9% in poetry books. Therefore, most respondents (30.1%) were interested in novels. The studied sample description in terms of “mental health”.

**Figure 2.**
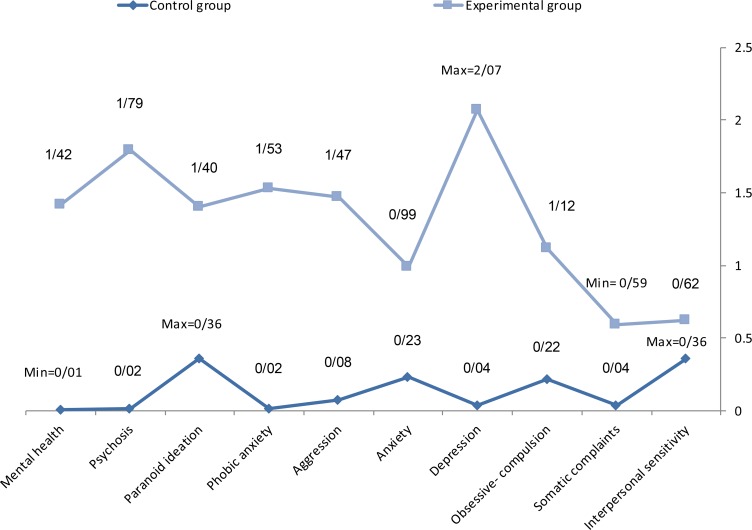
Difference between the control and experimental groups.

According to Table 3, the median and mean estimated values for the aspects of “mental health” in both control and experimental groups based on the pretest and posttest are very close together. Therefore, the scores of “mental health” in both groups have normal distribution according to the pretest and posttest results. Also, in both groups, scores of “mental health” in the samples are homogenous. The difference between pretest and posttest scores of “mental health” in the experimental group is 1.24 and pretest and posttest mean difference of “mental health” in the control group is −0.01. The differences between pretest and posttest scores of research variables in the control and experimental groups are presented in Table 4 and Figure 2. As seen in Table 4, the difference between mean scores of posttest and pretest in the control group is very low (less than 0.5).

**Table 3.** Statistics of “mental health” variables.

**Statistical Indicators**		**Pretest**		**Posttest**	

		**Control Group**	**Experimental Group**	**Control Group**	**Experimental Group**
Number of questions		90	90	90	90
The central tendency measures	Mean	1.87	1.90	1.86	3.32
	Median	1.83	1.83	1.83	3.27
	Mode	1.14	1.12	1.35	2.75
	Total	56.26	57.09	56.01	99.76
Dispersion measures	Standard Deviation	0.373	0.399	0.294	0.316
	Lowest observation	1.14	1.12	1.35	2.75
	Most observation	2.49	2.53	2.63	4.15
	Variation range	1.35	1.41	1.27	1.40
Distribution measures	Skewness	−0.045	−0.090	0.693	0.644
	Deviation of skewness	0.427	0.427	0.427	0.427
	kurtosis	−0.888	−0.886	0.438	0.509
	Deviation of kurtosis	0.833	0.833	0.833	0.833

**Table 4.** The difference between pretest and posttest scores of the research variables in the control and experimental groups.

**Research Variables**	**Control Group**	**Experimental Group**	**Absolute Value of Difference**
Interpersonal sensitivity	0.36	− 0.62	0.98
Somatic complaints	0.04	− 0.59	0.63
Obsession-compulsion	0.22	− 1.12	1.34
Depression	− 0.04	− 2.07	2.03
Anxiety	0.23	− 0.99	1.22
Aggression	− 0.08	− 1.47	1.39
Phobic anxiety	0.02	− 1.53	1.55
Paranoid ideation	0.36	− 1.40	1.76
Psychosis	− 0.02	− 1.79	1.77
Mental health	− 0.01	1.42	1.43

**Figure 3.**
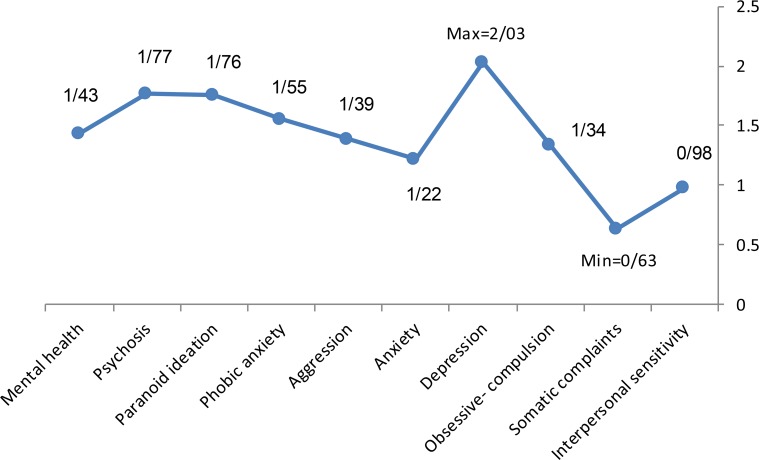
Mean difference between pretest and posttest scores of the research variables in control and experimental groups.

Therefore, there is no significant difference between mental health and its dimensions in the elderly people who did not participate in audio book workshops, but the difference between mean scores of posttest and pretest scores in the experimental group were significant (more than 0.5). In addition, this difference in the aspects of mental health in the experimental group is negative. Therefore, participation in audio book workshops reduces dimensions of mental health in the elderly, and this difference in “mental health” in the experimental group is positive. Thus, participation in audio book workshops has improved the mental health status of the old people. This difference between the control and experimental groups is presented in Figure 2. Based on the Table 4 data, the mean scores difference between pretest and posttest of the research variables in the control and experimental groups is shown in Figure 3. As seen in Figure 3, participation in audio book workshops reduces 98% interpersonal sensitivity, 63% somatic complaints, 1.34% obsession-compulsion, 2.03% depression, 1.22% anxiety, 1.39% aggression, 1.55% phobic anxiety, 1.76% paranoid ideation, 1.77% psychosis and 1.43% mental health of the elderly. Therefore, participation in audio book workshops has the greatest impact on “depression” and lowest effect on “somatic complaints” of the elderly.

### 3.2. Test research hypotheses

According to the verification prospectus of being independent groups, normal distribution of observations and significant difference between pretest and posttest scores, the Independent samples t test is used to compare posttest scores of the mental health and its dimensions of the control and experimental groups (Main research hypotheses). First research hypothesis: The mental health status of the elderly population significantly changed after participation in the workshops of audio books.

The significance level of Levene’s test by assuming equal variances of posttest scores of mental health and its dimensions in the control and experimental groups is more than 0.05. Therefore, assuming equal variances, posttest mean scores of mental health and its dimensions were compared between both control and experimental groups.

As seen in Table 5, the significance level of test comparison of posttest mean scores of mental health and its dimensions in both control and experimental groups is less than 0.05. Therefore, posttest scores mean of mental health and its dimensions in both control and experimental groups is accepted with 95% confidence level. Also, the rate calculated for different means of mental health dimensions is negative, so posttest mean scores of mental health dimensions in experimental group are less than the control group. This means that participating in audio book workshops decreased the mental health dimensions scores of the elderly. The mean difference of “mental health” is a positive number, so the posttest scores mean of mental health in the experimental group is more than the control group which means participating in audio book workshops increased “mental health” in the elderly. According to these results, the first hypothesis is confirmed with 95% confidence. Therefore, a significant difference is seen in the mental health and its dimensions of the elderly after participation in the audio books workshops. Second research hypothesis: The effect of audio books workshops participation on the mental health dimensions of the elderly is significant.

**Table 5.** Test results comparing the mean posttest results in both control and experimental groups.

	**Test Statistics**	**df**	**Mean Difference**	**Mean Difference Deviation**	**95% Confidence Interval**	
					**Lower Bound**	**Upper Bound**
Interpersonal sensitivity	−4.613	58	−0.882	0.191	−1.265	−0.499
Somatic complaints	−3.403	58	−0.600	0.176	−0.953	−0.247
Obsessive-compulsion	−9.046	58	−1.293	0.143	−1.580	−1.007
Depression	−8.406	58	−2.111	0.251	−2.614	−1.608
Anxiety	−6.148	58	−1.381	0.224	−1.830	−0.931
Aggression	−9.269	58	−1.469	0.158	−1.786	−1.151
Phobic anxiety	−8.166	58	−1.590	0.194	−1.980	−1.200
Paranoid ideation	−8.708	58	−1.991	0.228	−2.449	−1.533
Psychosis	−8.286	58	−1.805	0.217	−2.241	−1.369
Mental health	18.476	58	1.458	0.078	1.300	1.616

As it can be seen in Table 6, the significance level of comparison test of posttest scores rank mean of mental health dimensions in the experimental group is less than 0.05. Therefore, posttest mean scores of mental health dimensions in the experimental group is accepted with 95% confidence i.e., the effect of participation in audio books workshops on the dimensions of mental health of the elderly population was significant. The greatest impact of participation in the audio books workshops on the dimensions of mental health of the elderly population based on the average scores and from the highest to the lowest ones are paranoid ideation, psychosis, phobic anxiety, aggression, depression, interpersonal sensitivity, anxiety, obsession-compulsion, and somatic complaints.

**Table 6.** Friedman test results of experimental group’s posttest.

**Interpersonal Sensitivity**	**Mean Rank**	**Rank**
Somatic complaints	3.93	6
Obsessive-compulsion	3.63	9
Depression	3.03	8
Anxiety	5.13	5
Aggression	3.50	7
Phobic anxiety	5.43	4
Paranoid ideation	6.30	3
Psychosis	8.23	1
Mental health	7.80	2
Observation number=30 Teststatistics=155.	Degree of freedom=8 Significance level=0.000	

Because the second research hypothesis is confirmed with 95% confidence, the impact of participation in the audio books workshops on the dimensions of mental health of the elderly population was significant.

## 4. Discussion

This study was conducted to investigate the impact of book and bibliotherapy on the mental health of the retired elderly people. However, most of the study population had no desire to cooperate and even refused to answer questions of the researchers. In addition, audio book workshops should be held in a suitable place tailored for physical and mental condition of the elderly. Thus equipping the workshop was our main challenge to hold courses, which should be provided in a quiet environment with comfortable seats so that old people feel comfortable and relaxed at least for one hour.

Studies indicated that the mental health status of these people before reading books was low and after the completion of reading books sessions, the elderly’s mental health status improved to a relatively favorable level. According to information obtained, the participation in the audio books workshops had the greatest impact on the dimensions of paranoid ideation, psychosis, phobic anxiety, aggression, depression, interpersonal sensitivity, anxiety, obsession-compulsion, and somatic complaints, from the highest to the lowest. It seems that bibliotherapy and book reading to the elderly can improve the old age diseases such as depression. Since the use of audio books for mental health is considered a kind of bibliotherapy, the studies that evaluated the impact of bibliotherapy on the mental disorders and symptoms associated with their mental health, are also taken into consideration. The findings of the current study correspond with other related studies.

Suki et al. (2011) in their study examined the role of the recitation of Quran on mental health. They reported significant relationship of Quran recitation on mental health and introduced it as the most effective predictor factor on the mental health of elderly. These findings supported the results of the present study. Sotoodehnavroodi considered story telling in groups for the elderly as an alleviating factor of physical symptoms, depression and anxiety that was consistent with current research findings (Sotodeh, Pooragha Roodbordeh, Kafi, Poornesaii, 2013). Riyahinia et al. (2014) have also indicated the effects of bibliotherapy on reducing depression. Also Gholizdeh et al. study that was done similar to the way of the present study confirmed the use of bibliotherapy as an improvement factor of self-management capabilities to enhance the quality of life and active presence in the community (Gholi Zadeh, Khankeh, Mohammadi, 2011).

Mosharraf et al. also confirmed the positive impact of bibliotherapy on psychological features corresponding to the components studied in this research (Mosharraf, Papi, Zare Farashbandi, Samouei, & Hasanzadeh, 2015) which was consistent with the obtained results. Tajdaran et al. showed the favorable effects of bibliotherapy on increasing optimism in adolescents, so their results are in line with our study results (Tajdaran, Peyvastegar, & Moradi, 2014). Nabavi et al. reported that the higher social protection of the elderly can have a significant impact on their mental health and social functioning (Nabavi, Alipoor, Hejazi, Rabbani, & Rashedi, 2014). Therefore, in strategic planning, the elderly’s needs should be considered. Frieswijk et al. considered bibliotherapy as a factor that increases the ability of self-management in the elderly (Frieswijk, Steverink, Buunk, & Slaets, 2006). Lang also introduced audio books to maintain a positive feeling and reducing social isolation for the visually impaired elderly. Their findings are in line with this study result (Lang & Brooks, 2015). Floyd et al. compared the individual psychotherapy to bibliotherapy in the elderly with depression and preferred individual psychotherapy over bibliotherapy in the next stage of depression treatment, but considered bibliotherapy as an effective treatment of depression in the elderly, especially those who have no desire to engage in psychotherapy (Floyd, Scogin, McKendree-Smith, Floyd, & Rokke, 2004). Eum, Yim and Choi indicated that bibliography is a basis for literary treatment and can also heal the diseases of elderly such as dementia and depression that this result is in line with the current study result (Eum, Yim, & Choi, 2014). The results of this study are also consistent with the results of Joling et al. that studied the impact of bibliotherapy on the elderly on the verge of depression (Joling et al., 2011). Whittingham et al. also in their reviews have confirmed the positive effects of audio books on reading skills and a positive attitude to reading (Whittingham, Huffman, S., Christensen, R., & McAllister, 2013).

Reeves considered the bibliography as the effective quantity of self-medication in controlling stress and anxiety from mild to moderate (Reeves, 2010). Tukhareli introduced the bibliography as a factor influencing mental health and addressing the concerns and behavioral and physical problems. He suggested the bibliography as a widely recognized method to help librarians monitor people’s concerns or social, behavioral, physical, mental and emotional problems (Tukhareli, 2011). Murayama et al. asked the elderly to read illustrated books for school children. The results showed that intergenerational programs like reading decreases the risk of social isolation and loneliness in the elderly (Murayama et al., 2015). As indicated in this study, audio books affected the mental health components and thus its results are in line with this research.

Regarding that our country has already moved toward aging and old people’s needs must be attended to, it is expected that the obtained results be used by policy makers and health and social planners to improve the elderly’s health status. Authorities of the public health can allocate facilities and budget to the health sector, to improve the mental health of people, especially the elderly. Using reasonable and cost-effective solutions which are natural too for society can be receptive by community. Also a strategic approach in dealing with this issue prevents future social and cultural problems. Talking and audio tools are some of the least expensive ways to protect the health of people. The advantages of bibliotherapy are saving costs and time as well as the welfare of the elderly.

The role of books and reading in improvement of life and useful spending of leisure time is undeniable. However, the elderly for some reasons such as poor vision, impatience, and so on are less likely to use books and reading, while the book can be the best companion and can solve many psychological problems like depression, regretfulness, and so on. Text of books, on a sound device is the best choice for the elderly to improve their lives and protect public health. The advent of audio books after in print and electronic books is a manifestation of the technology for individuals. In order that the elderly enjoy the benefits of reading, authorities can plan the reading programs for them. It can gradually create a culture of reverence for the elderly in the family, intimacy in public as well as promoting the reading culture, especially among the family members. The more elderly people of society, as treasures of experience, become healthier and joyful, the more energetic will be the society.

Book reading requires institutionalization in the society and the internalization as a habit at the individual level. Promotion of ethical principles is certainly an important objective of the study that we follow from an early age (through school and family) to adulthood. If we accept that taking care of body, spirit, emotions, religion and spirituality is a crucial responsibility, and that each of us as a small unit of the community has commitment and responsibility towards the social system, then by choosing the right resources and spending time and sufficient energy, it would also be moral responsibility for them (Zokaei, 2011). Also, to improve the readings culture in our country, especially in the field of audio books and particularly at the level of the Internet, we need more effort and activity. E-books, electronic publishing, and audio books are new and innovative practices that publishers should be taken into consideration.
